# Adult onset Kawasaki disease presenting with acute epiglottitis findings^☆^

**DOI:** 10.1016/j.bjorl.2017.09.001

**Published:** 2017-10-05

**Authors:** Daichi Murakami, Gen Sugita, Mehmet Gunduz, Tomohiro Suenaga, Takashi Takeuchi, Hiroyuki Suzuki, Muneki Hotomi

**Affiliations:** a Wakayama Medical University, Department of Otorhinolaryngology-Head and Neck Surgery, Wakayama, Japan; b Wakayama Medical University, Department of Pediatrics, Wakayama, Japan

## Introduction

Kawasaki disease (KD) is a mucocutaneous lymph node syndrome, and was first described in Japan in 1967.^1^ Physiopathologically, it is an acute necrotizing vasculitis of the medium- and small-sized arteries. According to surveillance research from 2013 to 2014 in Japan, the sex ratio (male/female) was 1.28. KD often occurs in children and adult cases are very rare. The incidence rate (from 0 to 4 years old) was 305.3 per 100,000, while it is only 2.8 per 100,000 in those over 10 years of age. The ratio of patients under 3 years old was 63.5%. According to a recent review in 2015, only 100 cases of adult-onset KD had been described.^2^ The diagnosis of classical KD is made clinically with a fever of more than 5 days combined with at least 4 of the 5 clinical manifestations, including: painful erythematous or edematous changes in extremities especially in hands, polymorphous exanthema, erythematous changes in oral or pharyngeal mucosa or cracking of lips, painless and nonpurulent conjunctival injection and cervical lymphadenopathy. There is also an incomplete form of KD appearing with fever and some of the above clinical findings. Supportive laboratory changes such as leukocytosis, increased CRP, anemia, and hypoalbuminemia are also seen. Importantly, incomplete KD should not be confused with atypical KD. While the former presents with few of the classical KD symptoms, the latter is admitted to the clinics with unusual symptoms such as facial nerve palsy, sensorineural hearing loss or hepatic enlargement with jaundice. Some of the rarely seen cases are those admitted into hospitals with otolaryngological symptoms. Herein we report an adult-onset KD patient, who was initially admitted with acute epiglottitis.

## Case report

### Clinical history

**Figure 1:**
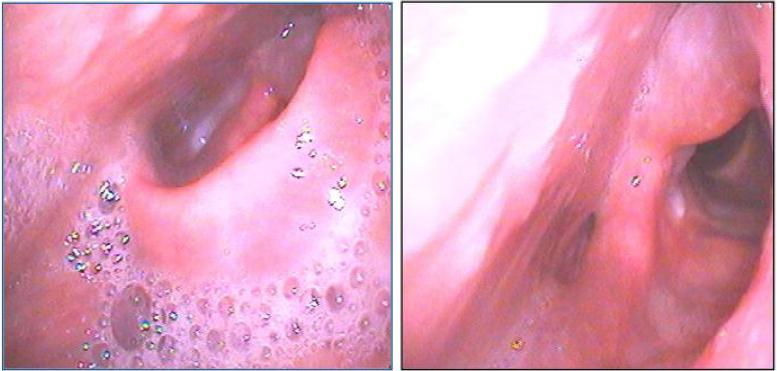
Laryngeal fiberscope at admission. Epiglottis and left arytenoid mucosa are edematous.

A 35-year-old Japanese man without any previous health problems was admitted to a local otolaryngology clinic with high fever of 39 °C, sore throat and odynophagia. Because laryngeal fiberscope showed swelling of his epiglottis, aryepiglottic fold and left arytenoid mucosa (Fig. 1), he was initially diagnosed to have acute epiglottitis. For airway management, he was transferred to our Medical University Hospital. We initially started a treatment by intravenous drip infusion of Sulbactam/Ampicillin (SBT/ABPC) 9 g/day and hydrocortisone 500 mg/day.

### Laboratory findings

On admission, his temperature, blood pressure and pulse were 39.4 °C, 103/53 mmHg, and pulse 98 min, respectively. Laboratory tests disclosed leukocytosis at 20 × 10^9^/L with predominance of polymorphonuclear cells (88.3%) and C-Reactive Protein (CRP) level was 24.0 mg/dL.

## Admission note

**Figure 2:**
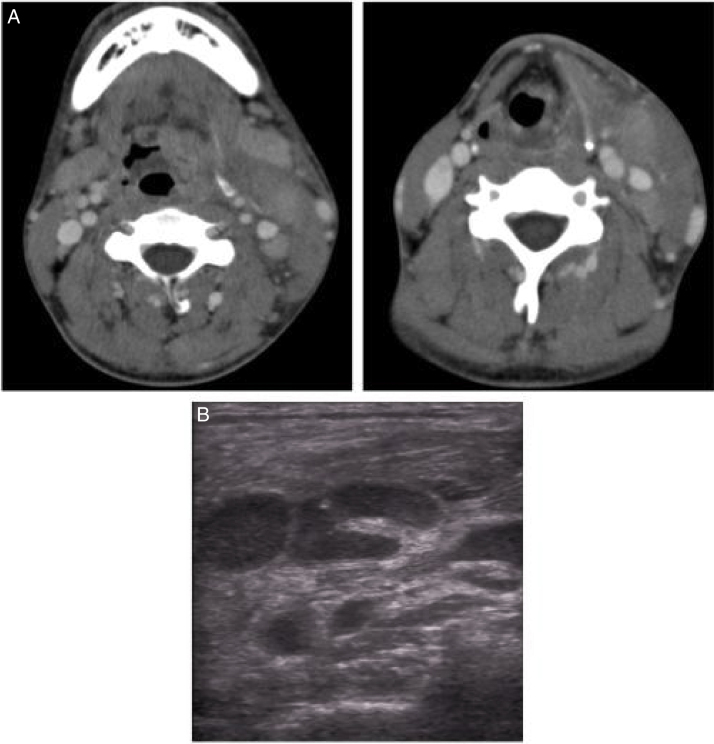
Cervical ultrasonic examination and enhanced computerized tomography (A, B). (A) Ultrasonic examination shows swellings of cervical lymph nodes. (B) Enhanced computed tomography shows swellings of cervical lymph nodes and edema of soft tissue and sternocleidomastoid muscle.

On day 2 of hospitalization, he was noted to have left cervical swelling. Cervical ultrasonic examination and enhanced computerized tomography (CT) showed left cervical lymph node swelling (Fig. 2) with edema of soft tissue and sternocleidomastoid muscle. We suspected phlegmon caused by cervical lymphadenitis and changed the antibacterial drug to Panipenem/Betamipron (PAPM/BP).

Although edema and mucosal swelling of the larynx gradually improved, fever continued to be high. Serologic tests were negative for antistreptolysin-O, herpes simplex virus-1, varicella-zoster virus, Epstein–Barr virus, cytomegalovirus, parvovirus B19, human T-cell lymphotropic virus, human immunodeficiency virus, toxoplasmosis and tuberculosis. Laboratory tests showed persistence of a high level of WBC, CRP and elevation of ferritin. The result of fine needle aspiration cytology from neck lymph node was CLASS III without any definitive diagnosis.

**Figure 3:**
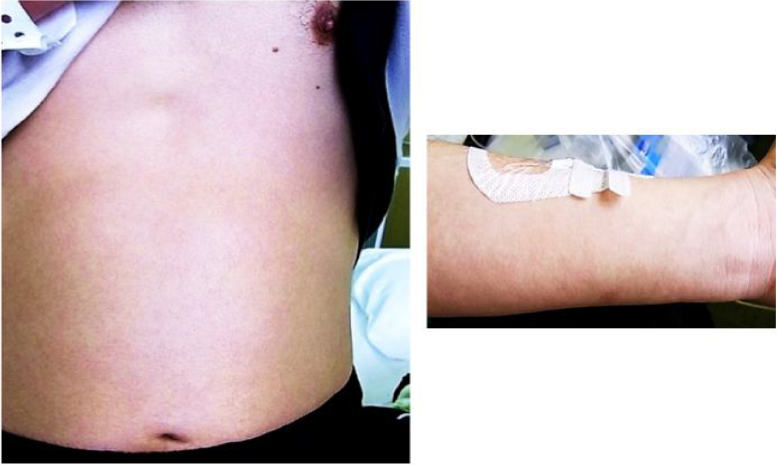
Erythematous rash at abdomen and extremities.

We performed open biopsy of cervical lymph nodes and attempted drainage. However, there was no pooling of pus. The result of biopsy was atypical lymphoproliferative lesion, and the result of bacterial culture was *Staphylococcus capitis subspieces ureolyticus* (+). On day 5 of hospitalization, he developed an erythematous rash of his abdomen and extremities (Fig. 3), and elevation of hepatic enzymes such as AST, ALT or γ-GTP was seen. His physical findings suggested severe infection caused by resistant bacterium (especially by Methicillin-Resistant *Staphylococcus aureus* – MRSA) or drug eruption and drug-induced hepatitis. We changed PAPM/BP to SBT/ABPC to avoid drug eruption and drug-induced hepatitis, and added oral Linezolid (LZD) to treat MRSA and intravenous immunoglobulin (IVIg) to treat severe bacterial infection. Because swelling of the laryngeal mucosa was improved, administration of hydrocortisone was ceased.

Despite various treatment protocols, high fever and cervical swelling persisted and laboratory findings revealed an increase of WBC and CRP levels. On day 7 of the hospitalization, he developed bilateral conjunctival injection, diagnosed as uveitis. Resistance to antibacterial treatment as well as the diagnosis of uveitis suggested that he had nonsuppurative inflammatory diseases. Bacterial cultures including blood, urine and sputum were negative and beta-D glucan was not elevated, so we concluded that he did not have infection disease. Rheumatoid factor, antinuclear antibody and anti-neutrophil cytoplasmic antibodies were all negative, but his findings suggested adult Still disease. Clinical criteria of adult Still disease are as follows:

Major criteria: (1) fever of 39 °C or higher (>1 week); (2) arthralgia (>2 weeks); (3) Salmon colored masculopapular rash; (4) leukocytosis (>10,000 μL with >80% granulocytes).
Minor criteria: (1) sore throat; (2) lymphadenopathy and/or splenomegaly; (3) liver dysfunction; (4) Negative Rheumatoid Factor (RF) and antinuclear antibody (ANA) test. Five features of Yamaguchi criteria, including at least two major criteria, must be present for adult onset Still disease diagnosis.^3^ Methylprednisolone pulse therapy (1000 mg/day) was initiated.

**Figure 4:**
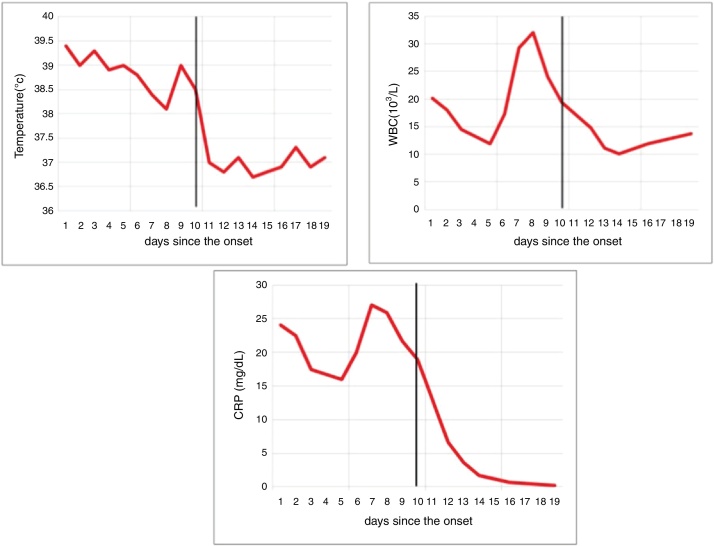
Clinico-laboratory data before and after methylprednisolone pulse. The black vertical line indicates the methylprednisolone pulse treatment event.

After initiation of methylprednisolone pulse therapy, he improved over the next 48 h, and WBC and CRP levels were decreased (Fig. 4). Cervical lymphadenopathy, erythematous rush and conjunctival injection were also improved.

**Figure 5:**
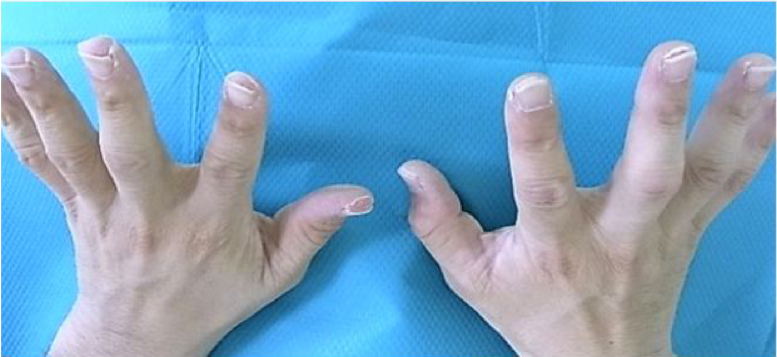
Fingertips desquamation.

At day 15 of hospitalization, his fingertips began to desquamate (Fig. 5). In addition to desquamation, high fever, cervical lymphadenopathy, erythematous rush and conjunctival injection suggested Kawasaki disease. We consulted with the department of pediatrics, and a clinical diagnosis of Kawasaki disease was made. An echocardiogram showed no coronary aneurysm. From day 18 of hospitalization, intravenous methylprednisolone was changed to oral prednisolone, and the dose of prednisolone was decreased. Finally, oral prednisolone use was discontinued and the patient was discharged.

## Discussion

In this report, we present a patient with rarely seen adult-onset Kawasaki disease (AKD) case with initial findings of acute epiglottitis. A complete diagnosis of KD is defined by the presence of fever of 5 days or more without any other specific explanation and at least 4 of the 5 following criteria: (1) polymorphic exanthema; (2) changes in peripheral extremities; (3) bilateral nonexudative conjunctival injection; (4) changes in the oropharyngeal mucosa; (5) acute nonsuppurative cervical lymphadenopathy.^4^ Incomplete KD is defined by 3 of the above 5 criteria and coronary artery disease. AKD is defined as KD onset after the age of 18. AKD is rare and often misdiagnosed.

There are only 2 cases reported of KD patients presenting with acute epiglottitis in the literature, but they were children (13 years old and 9 years old).^5^ This report is the first of an AKD patient presenting with findings of acute epiglottitis.

The patient with KD shows high fever, inflammatory oropharyngeal changes, lymphadenopathy with elevations of inflammatory signs such as WBC and CRP levels, and symptoms might not present simultaneously, so the diagnosis of infectious diseases is often made. Despite administration of antibiotics, the symptoms do not improve, so other diagnoses (for example, drug hypersensitivity syndromes, toxic shock syndrome or autoimmune diseases such as adult Still disease) are often suggested. In fact, when the patient was admitted, we made a diagnosis of bacterial phlegmon caused by cervical lymphadenitis and then adult Still disease.

Other otolaryngological symptoms such as peritonsillitis or retropharyngeal abscess in Kawasaki disease have also been reported.^6^, ^7^, ^8^, ^9^, ^10^ Coronary artery damage is the main fatal complication of Kawasaki disease, so delay of diagnosis or treatment of Kawasaki disease sometimes leads to coronary infarction or sudden death. Fortunately, our patient did not have any coronary abnormality.

This case highlights that when we face a patient with otolaryngological infection who does not respond to antibiotics therapy, we need to discuss the possibility of Kawasaki disease, even if the patient is an adult.

## Conclusion

There are rare cases of Kawasaki disease which may present with various otolaryngological infection-like symptoms, even if they are adults. When the response to antibiotics is poor, otolaryngologists should consider the possibility of Kawasaki disease to avoid the delay of diagnosis and formation of coronary abnormalities.

## Conflicts of interest

The authors declare no conflicts of interest.
